# Recurrent pneumonia with persistent peripheral eosinophilia: Idiopathic chronic eosinophilic pneumonia

**DOI:** 10.1002/ccr3.4653

**Published:** 2021-08-15

**Authors:** Graey Wolfley, Francis Essien, John Untisz

**Affiliations:** ^1^ Department of Internal Medicine Keesler Air Force Base Keesler Medical Center Biloxi Mississippi USA; ^2^ Department of Pulmonology/Critical Care Keesler Air Force Base Keesler Medical Center Biloxi Mississippi USA

**Keywords:** chronic eosinophilic pneumonia, ground‐glass opacities, hypoxia, persistent eosinophilia

## Abstract

Although ICEP is an exceptionally rare disease, it is potentially overlooked and misdiagnosed. This case highlights the importance of peripheral eosinophilia in raising suspicion for ICEP. Without treatment, majority of patients fail to improve.

## Graphical abstract

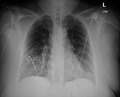

## 1 INTRODUCTION

Idiopathic chronic eosinophilic pneumonia (ICEP) is a rare pulmonary disorder. Due to the broad differential of diseases with similar symptoms and imaging findings, ICEP can be mistaken for infectious etiologies. The presence of peripheral eosinophilia in addition to history should raise suspicion for ICEP.

Idiopathic chronic eosinophilic pneumonia, also known by the eponym Carrington disease, is a rare eosinophilic lung disease that accounts for less than 3% of cases of interstitial lung disorders.^1^ Patients present with ill‐defined symptoms of dyspnea, cough, wheezing, and fatigue over several months as well as the presence of eosinophilia and opacities on chest imaging.^2^, ^3^ Once diagnosed, ICEP typically responds well to corticosteroid therapy. However due to the broad differential of diseases with similar respiratory symptoms and imaging findings, ICEP can be mistaken for infectious etiologies. This leads to receiving inappropriate courses of antimicrobials and delays definitive treatment. It is essential to identify key components of the patient's history and presence of peripheral eosinophilia to raise suspicion for ICEP and pursue further testing to confirm the diagnosis. We present a case demonstrating a patient with ICEP heralded by significant peripheral eosinophilia that had been overlooked and misattributed to infectious etiologies on several encounters over the course of a year.

## 2 CASE REPORT

57‐year old Caucasian female with a remote smoking history who presented with complaint of dyspnea, productive cough and wheezing. Symptoms had been worsening over the prior 10 days, although she also described subtle worsening of dyspnea for several months. She had been seen on four separate occasions over the past year presenting with similar symptoms which were progressively worse on each occasion and never fully resolved. On each occasion, she was empirically treated for presumed bacterial pneumonia with antibiotics and short courses of oral steroids with symptomatic improvement. However, cough and dyspnea returned within a short period of time after completing steroid courses. The most recent episode was 1 month prior to admission, at which time she was again treated with course of antibiotics for suspected pneumonia, as well as a course of prednisone.

**FIGURE 1:**
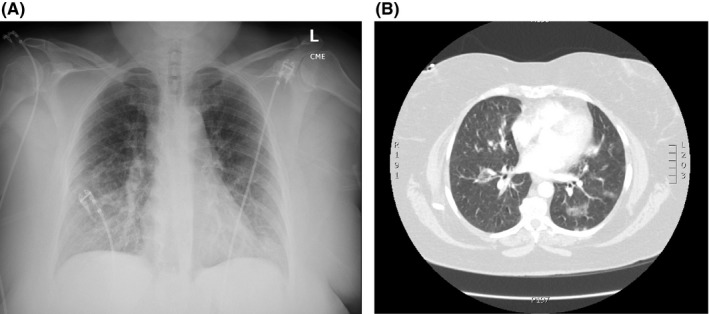
A, Portable chest X‐ray noting patchy interstitial and alveolar infiltrates. B, Chest CT‐Angiogram (Pulmonary embolism protocol) with multiple peripherally based groundglass multilobar opacities

On presentation, she was hypoxemic with an O_2_ saturation of 62% on room air, visibly dyspneic with diffuse wheezing throughout lung fields on auscultation. Laboratories were notable for leukocytosis (WBC 16.34 × 10^3^/mcl) and eosinophilia (14.4%, absolute 2.4 × 10^3^/mcl). Inflammatory markers were elevated with a CRP of 5.95 mg/dl and an ESR of 52 mm/h. Review of prior laboratories similarly noted presence of eosinophilia over the past several years. Plain film chest imaging (Figure 1A) noted patchy interstitial alveolar opacities. Contrasted CT chest (Figure 1B) was remarkable for multilobar, peripherally based ground‐glass opacities. She was admitted for acute hypoxic respiratory failure secondary to suspected eosinophilic pneumonia.

History and review of current medications did not identify a culprit agent for eosinophilic pneumonia. Infectious and vasculitis workups for alternative causes of eosinophilic pneumonia including ANA, c‐ANCA, p‐ANCA, rheumatoid factor cyclic citrullinated peptide (CCP), strongyloides antibodies, aspergillus antibodies, COVID PCR, Influenza PCR, and RSV PCR were negative. IgE testing resulted in an elevated level at 643.3 IU/ml. The patient underwent and urgent bronchoscopic evaluation after consultation with pulmonology. Bronchoscopy with bronchoalveolar lavage (BAL) was performed in the right middle lobe, most notable for negative cultures and a cell differential of 58% eosinophilia. Given her clinical presentation, peripheral eosinophilia, >25% eosinophilia on BAL, and radiographic opacities, the diagnosis of chronic eosinophilic pneumonia was made.

High dose IV methylprednisolone at 125 mg every 6 h was started with near immediate symptomatic improvement. She was transitioned to oral prednisone 50 mg daily (0.5 mg/kg), and she was eventually weaned off supplemental oxygen. She was discharged with 3‐month prednisone taper rather than 6 months due to her being a type 2 diabetic. She has continued to do well post‐discharge with peripheral eosinophilia down to 0.1 × 10^3^/mcl.

## 3 DISCUSSION

Epidemiologically, ICEP more commonly affects women compared to men with the mean age of 45 years at the time of diagnosis.^1^ Traditionally, ICEP presents with respiratory complaints of cough, dyspnea, and wheezing. Systemic symptoms of fatigue, fever, malaise, and weight loss are also often present. Leukocytosis and peripheral eosinophilia are present in more than 90% of cases and may help signify an alternative diagnosis other than bacterial/viral pneumonia. There are no absolute diagnostic criteria for ICEP but diagnosis is made based on respiratory symptoms for at least 2 weeks, presence of multilobar peripheral ground‐glass opacities, and eosinophilia >40% on BAL or peripheral eosinophilia >1000/mm^3^ in the absence of other eosinophilic lung diseases.^1^, ^3^ Other such causes of eosinophilic pneumonia that need to be excluded prior to diagnosis include but not limited to drug‐induced eosinophilic pneumonia, eosinophilic granulomatosis with polyangiitis (EGPA, also known as Churg Strauss), allergic bronchopulmonary aspergillosis (ABPA), and fungal and parasitic infections depending on exposure history.^3^

Once diagnosed and treatment is started, patients typically respond well to steroids with improvement in symptoms and rapid resolution of eosinophilia. However, biologics also have a role in cases refractory to corticosteroids^2^, ^4^ or as steroid‐sparing agents.

In our patient, other causes for eosinophilic pneumonia were excluded and the diagnosis of ICEP was made based on the aforementioned criteria with the patient improving significantly once started on steroids. Had her peripheral eosinophilia and history of recurrent pneumonia been overlooked, it is likely that the diagnosis of ICEP would not have been made.

This case demonstrates that although ICEP is an exceptionally rare disease, it is potentially overlooked and highlights the importance of peripheral eosinophilia in raising suspicion for IECP. Without treatment, <10% of patients spontaneously improve. Therefore, it cannot be overstated that a thorough history and physical in addition to radiographic and serologic assessment all the while maintaining a broad differential diagnosis allowed for the diagnosis of ICEP in this patient.

## CONFLICT OF INTEREST

The authors have no potential conflict of interest in regards to the contents of this manuscript.

## AUTHOR CONTRIBUTIONS

GW involved in writing article and literature search. FE treated patient and involved in literature search, reviewing and assisting with article preparation. JU treated patient and involved in supervision, reviewing and editing.

## ETHICAL APPROVAL

No formal ethical board approval was required for this case report.

## INFORMED CONSENT

Informed consent was obtained from the patient for their anonymized information to be published in this article.

## Data Availability

Data sharing not applicable to this article as no datasets were generated or analyzed during the case report.
